# Plasmapheresis for extracorporeal membrane oxygenation (ECMO)-induced hemolysis in infants

**DOI:** 10.1051/ject/2024032

**Published:** 2024-12-20

**Authors:** Gail Budhu, Kaydeen Morris-Whyte, Alexandru R. Constantinescu

**Affiliations:** 1 Pediatric Residency Program, Joe DiMaggio Children's Hospital, Memorial Healthcare System 1005 Joe DiMaggio Dr. Hollywood FL 33021 USA; 2 Division of Pediatric Nephrology, Joe DiMaggio Children’s Hospital 1131 N35th Ave Hollywood FL 33021 USA; 3 Charles E. Schmidt College of Medicine at Florida Atlantic University 777 Glades Rd BC-71 Boca Raton FL 33431 USA

**Keywords:** Therapeutic plasma exchange (TPE), ECMO-induced hemolysis, Infants

## Abstract

*Background*: Intravascular hemolysis is a known complication of extracorporeal membrane oxygenation (ECMO). Characterized by elevated plasma-free hemoglobin (PFH), intravascular hemolysis is associated with cytotoxic effects leading to renal replacement therapy (RRT), longer ECMO runs, and mortality. Therapeutic plasma exchange (TPE) in tandem with ECMO was described as a therapy for various pathologic conditions, but there are no Extracorporeal Life Support Organization (ELSO) guidelines for the treatment of ECMO-induced hemolysis. We describe the use of TPE in the management of severe ECMO-induced hemolysis. *Methods*: Two-term neonates receiving veno-arterial (VA) ECMO developed severe PFH, with peak values over 500 mg/dL. TPE was performed in tandem with the ECMO circuit. Packed red cells were used to prime the TPE circuit, and citrate anticoagulation was added to establish the interface, which could not be achieved with existing heparin in the ECMO circuit. Therapy was completed with saline solution as a decoy for citrate, to avoid hypocalcemia and intracranial bleeding. Plasma volume was replaced by fresh frozen plasma (FFP). *Results*: In one patient PFH fell to 120 mg/dL, but rebounded to close to 500 mg/dL, only to stabilize between 210 and 300 mg/dL after the second TPE. He was liberated from ECMO, but could not survive a respiratory decompensation. The other patient’s PFH improved to 360 mg/dL after one TPE and continued to decline to 120 mg/dL over the ensuing days. Despite that improvement, care was withdrawn. *Conclusion*: TPE is effective in decreasing the burden of PFH and is well tolerated in tandem with ECMO, and a database of infants with ECMO-induced hemolysis needs to be created to assess the current practice and establish clinical guidelines for its most appropriate therapy.

## Overview/Introduction

Extracorporeal membrane oxygenation (ECMO) is a lifesaving modality, being used more frequently nowadays in patient management due to its significant advances in quality, convenience, and accessibility. One of the major complications is intravascular hemolysis, which contributes to higher morbidity and mortality rates [1]. This is characterized by an increase in plasma-free hemoglobin (PFH, in mg/dL), more frequently seen in children, to levels of 100–500 (in up to 47.3% (mild hemolysis), 500–1,000 in 12.6–23.6% (moderate hemolysis) and levels >1,000 mg/dL in 6.8–43.5% (severe hemolysis) [1], lactate dehydrogenase (LDH) or total bilirubin (TB) [2, 3]. Various factors have been identified to contribute to hemolysis in these patients, including the presence of thrombi within the circuit, high negative inlet pressure, excessive pump speed, shear stress exerted on red blood cells, and high-velocity flows through small cannulae [1, 2]. Additionally, oxygenator-related factors such as cavitation, pressure fluctuations, and the duration of ECMO support, also play a role [1–5]. The most frequently encountered cause of hemolysis is thrombosis of the pump head, with experts recommending replacing it or replacing the entire ECMO circuit as the initial step [6]. However, there are no Extracorporeal Life Support Organization (ELSO) guidelines for the treatment of ECMO-induced hemolysis. Most care in pediatrics is off-label, universally accepted guidelines do not exist for most of our treatments. The problem is even more challenging in infants due to their small size and increased risk of intracranial hemorrhage. The following two cases illustrate instances in which hemolysis occurred and was significantly reduced by therapeutic plasma exchange (TPE). Plasma exchange is a therapeutic procedure involving the selective removal of plasma volume (usually 1×), replaced with either albumin or fresh frozen plasma (FFP), depending on the specific indication for TPE [2]. It is being used in tandem with ECMO for a variety of disorders, such as rejection of transplanted organs, sepsis with multiple organ failure, thrombocytopenia-associated multiple organ failure, alveolar hemorrhage in polyangiitis with granulomatosis, acute lung injury from H1N1 infection, acute respiratory failure due to COVID-19 infection, or as a strategy to reduce the cytokine burden [7]. In both pediatric and adult patients, the tandem use of ECMO and TPE was associated with electrolyte abnormalities (most notably hypocalcemia) and coagulopathies, along with hemodynamic instability [7]. However, only case reports mention TPE as a form of therapy for ECMO-induced hemolysis [8]. The purpose of this case series is to present the use of TPE in severe ECMO-induced hemolysis.

## Description

### Case 1

**Figure 1:**
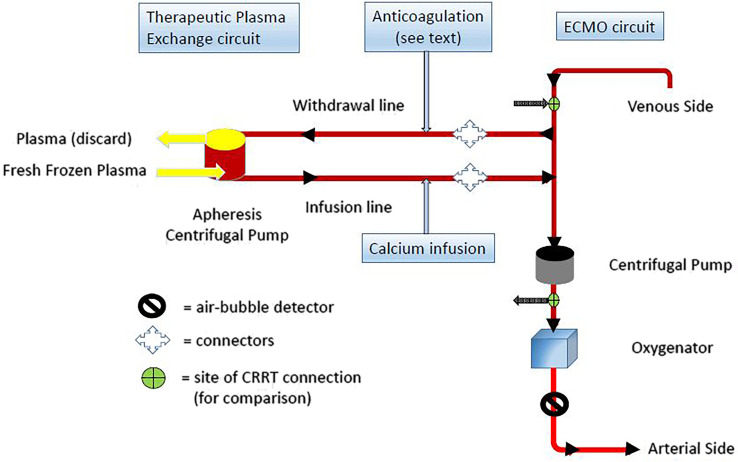
Connection of TPE circuit to the ECMO circuit.

A 7-day-old male neonate born at term by emergency C-section for fetal distress due to congenital diaphragmatic hernia and secondary respiratory decompensation, weighing 3.2 kg (birth weight 3.27 kg), was placed on ECMO, Rotaflow system with Quadrox-ID oxygenator (Maquet, Germany). He was cannulated peripherally (neck): V-10Fr, A-8Fr. The hemolysis became apparent, as evidenced by a steady rise in PFH (tested with HemoCue [9]), within a day of ECMO initiation. Revolutions per minute (RPM) was kept below 3,000 (average 2,000–2,600), and venous pressure average −9 (range −6 to −24). Other causes of hemolysis, not related to ECMO, were excluded (i.e., enzyme deficiencies, hemoglobinopathies, autoimmune hemolytic anemia, microangiopathies, etc). The oxygenator was changed, he underwent echocardiographic repositioning of cannulae, and because fibrinous deposits were noted, the ECMO circuit was exchanged (equivalent to an exchange transfusion), though it yielded no improvement in PFH, which peaked at 1,050 mg/dL. TPE using the Spectra Optia system (Terumo Medical Corporation, Tokyo, Japan) was performed in tandem with ECMO (see diagram of connection Figure 1).

Packed red blood cells (pRBCs) were used to prime the TPE circuit. Even though he received systemic heparin, the interface could not be achieved after 45 min. Therefore, we used an anticoagulation protocol previously described [10]. Citrate was added in a blood-to-citrate ratio of 15:1. After the interface was quickly established, citrate was replaced with a 0.9% NaCl decoy solution to complete the therapy about 2 h later, with a total of 4 mL of citrate. Plasma volume (1×) was replaced with FFP. Post-procedure ionized calcium was 1.26 mmol/L, normal range in our laboratory 1.16–1.45 mmol/L.

**Figure 2:**
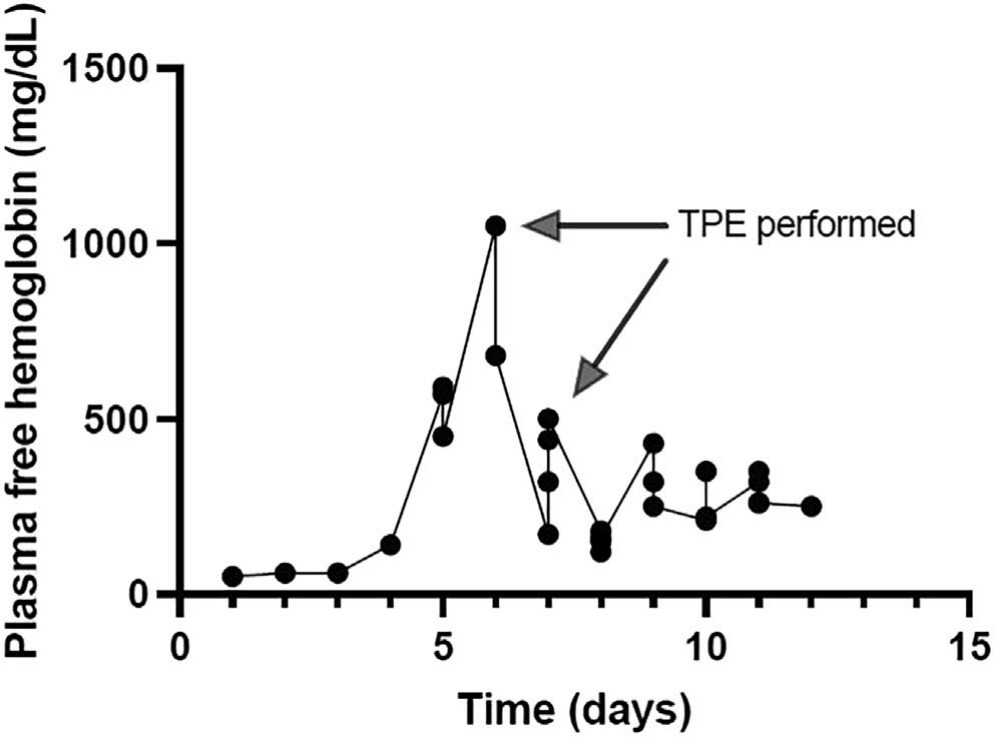
PFH trend for patient in case #1 who received two rounds of plasmapheresis.

After TPE, PFH levels fell immediately to 680 mg/dL and further down to 120 mg/dL during the ensuing hours. However, this improvement was short-lived as levels slowly increased close to 500 mg/dL (Figure 2) 2 days later. A second round of TPE was performed. Due to a higher hematocrit, treatment had a slightly longer duration. No hypotension or hypocalcemia were noted. After that second round, PFH dropped to 220 mg/dL. Levels were monitored closely and remained between 210 and 350 mg/dL. The patient was liberated from ECMO and de-cannulated two days after his second TPE, without sacrificing the carotid artery. He experienced respiratory decompensation, culminating in cardiac arrest before continuous renal replacement therapy (CRRT) could be started for new onset oligo-anuria. At the time of arrest, he did not meet the requirements for re-cannulation and did not survive.

### Case 2

A term male neonate, small for gestational age, with a birth weight of 2.52 kg, was diagnosed with hypoplastic left heart syndrome, mitral stenosis, and aortic stenosis. On DOL#3 underwent a modified Norwood procedure and coarctation resection. On DOL#4 developed pulmonary over-circulation and worsening lactic acidosis. Cardiac catheterization revealed an obstructive process at the level of the aorta. During cardiac catheterization, the patient experienced cardiac arrest, requiring prolonged resuscitation, followed by central cannulation (V-16Fr, A-8Fr) for V-A ECMO (Rotaflow system with Quadrox-ID oxygenator, Maquet, Germany). While receiving ECMO, PFH peaked at 620 mg/dL. The RPM was kept below 3,000 (range 2,190–2,255) and the venous pressures were between −25 and +10. TPE was initiated in tandem with ECMO (Figure 1). The previously mentioned causes of hemolysis were excluded in this case as well.

As in case #1, since the patient was already on heparin therapy, citrate was added as previously described. The patient received less than 5 mL of citrate during the treatment, which was completed in less than 2 hours. Plasma volume (1×) was replaced by FFP. No hypotension was noted and ionized calcium remained normal (1.33 mmol/L) during the procedure.

**Figure 3:**
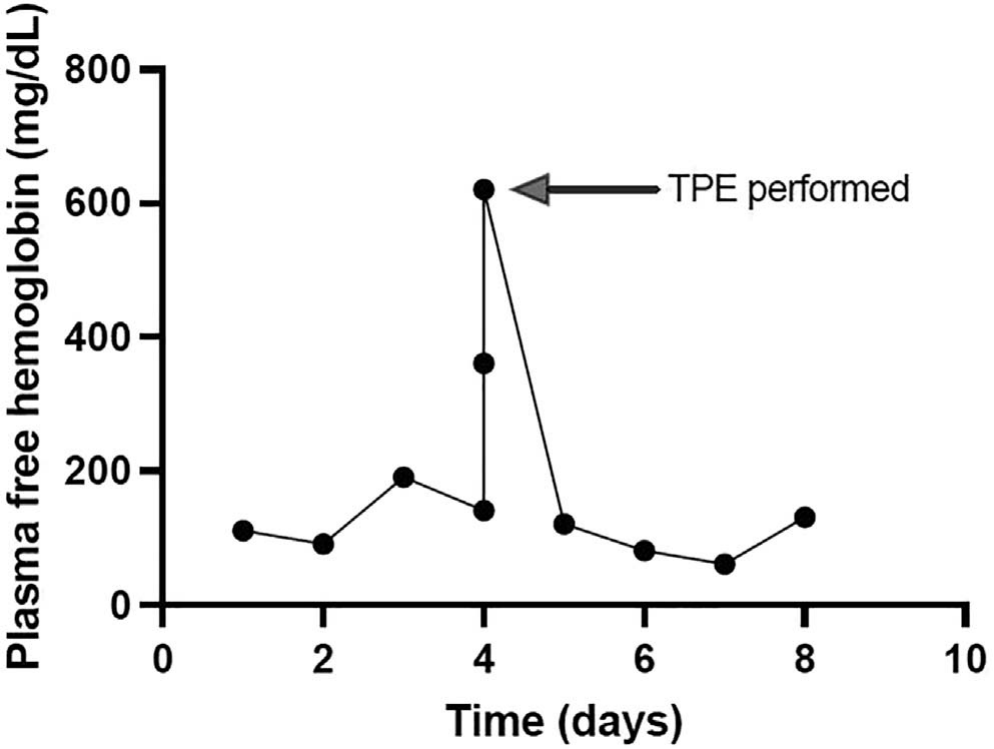
PFH trend for patient in case #2 who received one round of plasmapheresis.

Before TPE, PFH was 620 mg/dL. Immediately after, levels improved to 360 mg/dL and, in the ensuing days, to below 130 mg/dL (Figure 3). Unsuccessful attempts were made to liberate the neonate from ECMO due to hypotension and systemic hypoxemia on the background of severe tricuspid regurgitation. Ultimately, care was withdrawn at the parental request.

## Comment

Hemolysis is a life-threatening complication associated with extracorporeal life support (ECLS) [3]. The morbidity is mainly secondary to the byproducts of hemolysis, namely PFH. During ECMO, PFH levels can increase well above normal levels [11]. At such elevated levels of PFH, tissue hypoxia and cell death can occur [12]. High PFH levels are linked to multi-organ failure, including direct kidney injury [1, 3]. Moreover, PFH depletes vascular nitric oxide, resulting in peripheral vasoconstriction, inappropriate platelet activation, worsening ischemic injury, and increasing risk of thrombus formation [1, 2]. Some consequences of ECMO-induced hemolysis include increased requirement for blood products, need for CRRT, prolonged ECMO, implicit extended ICU stays, and increased mortality [1, 13, 14]. Despite several improvements aimed at mitigating hemolysis during ECMO [6], it remains a significant source of morbidity.

In our center, if the rate of PFH rises (doubling every 12 h) and/or levels of 500 mg/dL are noted, the oxygenator is changed first, then cannulae are checked and repositioned if needed, subsequently, the circuit change is performed, and if PFH does not decline, TPE is considered. This approach is similar to the reported strategies for decreasing ECMO-induced hemolysis, which include circuit replacement, whether partially or wholly, exchange transfusions (ET) – one to two times blood volume, peripherally or centrally – and plasma exchange – one to two times plasma volume [6, 15]. If these strategies fail, no immediate solution is available. In the cases highlighted, TPE showed early signs of benefit. The uniqueness is the use of TPE as first-line therapy in severe ECMO-induced hemolysis after ruling out other causes and visualizing the absence of thrombi or fibrin deposition in the ECMO circuit.

TPE has been employed in severe intravascular hemolysis [16] and is similar to ET in that it can reduce PFH and TB significantly. Early implementation has been shown to prevent acute kidney injury [15].

Plasma exchange in tandem with the ECMO circuit can exchange 1.5–2 times the estimated plasma volume [15]. Complications associated with TPE during ECMO include access malfunction, circuit complications (i.e., thrombosis), hypotension, and hypocalcemia. It is important to note that TPE can induce severe coagulopathy if albumin is used for replacement instead of FFP, requiring close monitoring of coagulation factors.

In the cases presented, TPE was set up with the typical anticoagulant, citrate [17] in the blood-to-citrate ratio of 15:1 to achieve interface sooner. Subsequently, citrate was replaced by 0.9% NaCl to minimize the risk of intracranial bleeding in already systemically heparinized patients. We also wanted to avoid hypocalcemia, a relatively frequent complication. In a study of 76 patients, out of which 53 were children, hypocalcemia was noted in 27.6% of patients [7]. Our approach led to an overall reduction in the average TPE time without significant coagulation derangement, and the total citrate received was kept at a minimum. The TPE resulted in a sustained reduction of PFH and, thereby, in hemolysis and morbid sequelae. Though clinical outcomes were poor in both cases, the desired effect of reduction in hemolysis was achieved (NB. data is accrued continuously).

Given the wide range of incidence of ECMO-induced hemolysis, we interrogated the 2023 ELSO report with respect to infants (defined as <28 days old) and found out that for the subpopulation with cardiac disease requiring ECMO, severe hemolysis (defined as PFH > 100) was reported in 54/366 cases (14.8%) with 28% survival rate. In turn, for those with respiratory illnesses requiring ECMO, in the same time period, there were 67/475 cases of severe hemolysis (14.1%), with a 43% survival rate.

We conclude that TPE plays a role in managing severe ECMO-induced hemolysis and is feasible to be used in tandem with ECMO in infants and older children. In the absence of randomized studies to date, a database of patients experiencing ECMO-induced hemolysis should be set up, with the scope of creating clinical practice guidelines for adequate therapy. After ensuring cannulae are in the first line of therapy for severe correct position and void of thrombi or fibrin deposition, we could recommend TPE as the first line of therapy for severe ECMO-induced hemolysis. We also emphasize the importance of continuous monitoring of hemodynamics, urine output, ionized calcium, and blood gases during the procedure.

## Data Availability

All pertinent data are included in the manuscript.
